# Analyzing the Causes and Frequency of Early Dental Implant Failure among Iranians: An Epidemiological Study

**DOI:** 10.1155/2023/2107786

**Published:** 2023-10-10

**Authors:** Majid Hosseini Abrishami, Farid Shiezadeh, Sahand Samieirad, Melika Mollaei, Farzaneh MohammadZadeh Mahrokh, Farzaneh Khosravi

**Affiliations:** ^1^Department of Oral and Maxillofacial Surgery, Mashhad University of Medical Sciences, Mashhad, Iran; ^2^Department of Periodontics, Mashhad University of Medical Sciences, Mashhad, Iran; ^3^Oral and Maxillofacial Diseases Research Center, Mashhad University of Medical Sciences, Mashhad, Iran; ^4^Student Research Committee, Faculty of Dentistry, Mashhad University of Medical Sciences, Mashhad, Iran; ^5^Department of Restorative Dentistry, School of Dentistry, Mashhad University of Medical Sciences, Mashhad, Iran

## Abstract

**Aim:**

The rate of early dental implant failure (DIF) has increased in recent years, though the risk factors associated with this primary failure remain unclear. This study aimed to determine the rate of early implant failure and identify contributing factors. It was conducted from March 2018 to 2020 in Mashhad, Iran.

**Method:**

This observational study examined the records of 983 implants from the Implant Department of Mashhad Dental School. Variables considered included age, gender, systemic diseases, smoking habits, implant type and size, and surgery-related factors. Data were analyzed using Chi-square, Mann–Whitney *U*, and Fisher exact tests in SPSS V22, with a *p*-value of 0.05 or less considered statistically significant.

**Result:**

Of the 983 implants, 42 (4.3%) experienced early failure. The study population consisted of 555 (56.5%) females and 428 (43.5%) males, with an average age of 49.34 ± 13.67 years. A significant correlation was found between surgical complications (e.g., fracture of implant fixtures and inferior alveolar nerve exposure) and implant loading time (Yes or No) with early DIF (*p*=0.05 and *p* < 0.01, respectively). However, no significant correlation was observed between early failure and factors such as age, gender, smoking habits, systemic diseases, implant dimensions, or manufacturer.

**Conclusion:**

Surgical complications and loading time may be the most critical factors contributing to early implant failure. Therefore, we suggest dentists pay attention to the mentioned factors in the surgical protocols and their relationship. Further prospective studies on risk factors that could affect early implant failure are needed.

## 1. Introduction

Dental implants have emerged as the most promising treatment option for addressing partial or complete edentulousness, ensuring a firm and long-term stable osseointegration [1, 2]. Despite the high success rate and average survival rate of implants, which are 89.7% and 94.6%, respectively, failures still occur [1, 3]. Dental implant failure (DIF) occurs in 5% of cases, resulting from a lack of primary implant integration due to fibrous scar formation and inflammation of the periodontal tissues (peri-implantitis); DIF can be categorized into early and late losses [4, 5]. Early failures, which occur more frequently than late ones, occur before prostheses are placed by the prosthetist (typically within 3–5 months after implant placement) and are associated with slow bone healing [1, 6, 7].

The remainder of the relevant report indicates that a dental implant is at risk of early failure if it exhibits mobility, pain during function, and bone loss more significant than half of the implant's length [8]. The success of dental implant therapy depends on various etiologies, including both patient-related and nonpatient-related factors [8]. Patient-related factors encompass smoking habits, alcohol consumption, age, osseous density, bone quality, underlying pathologies, and systemic diseases such as cardiovascular diseases, thyroid diseases, diabetes, bruxism, the oral microbiome, and host response [1–5, 7–9]. Nonpatient-related factors include dental implant design, length, width, prosthetic component, surgical techniques, and the quality of materials used [8].

Previous studies have suggested a significant association between early implant failure and factors such as patient age, gender, and systemic diseases [6, 7]. However, some studies found no connection between these variables (*p* ≥ 0.05) [4, 10]. Notably, discrepancies exist between studies reporting the incidence of early DIF, with one retrospective study in 2019 stating a rate of 4.4% [10] and another study reporting 3.4% [5]. One investigation identified implant placement in the posterior mandible as a significant risk factor for early DIF [5]. At the same time, other studies have shown a higher risk of primary implant failure when placed in the maxilla (*p* ≤ 0.05) [11, 12].

Understanding the etiologies and influential risk factors associated with early implant failure is crucial, as patients experiencing this issue may face dissatisfaction and the need for secondary surgeries [7]. Considering the limited research on this topic in Iran, the absence of a relevant study in the Mashhad Dental Clinic, and the numerous controversies mentioned above, the authors aim to determine the early failure rate and potential causes to help surgeons recommend the best treatment options for their patients.

This study examines the early failure rate of various types of dental implants placed in the implant department at the Mashhad Faculty of Dentistry. The research compares the early failure rates of all dental implant types based on the manufacturing company and their microdesign surface. It also investigates other factors related to early implant failure.

The null hypothesis of this study posits that parameters such as systemic diseases, smoking habits, implant size, surgery-related factors, and loading time contribute to early implant failure (*p* ≤ 0.05).

## 2. Materials and Methods

### 2.1. Study Design

This historical observational study occurred from March 2018 to 2020 in Mashhad, Iran. Before registration, all participants signed a consent document per the Helsinki Declaration principles. The researchers pledged to maintain the confidentiality of patients' information. The study protocol received ethical approval from Mashhad University of Medical Sciences (IR.MUMS.DENTISTRY.REC.1399.065). The STROBE (Strengthening the Reporting of Observational Studies in Epidemiology) guidelines were adhered to, and no interventions were conducted during this research. The study population comprised patients referred to the implant department of Mashhad University of Medical Science's dental clinic over 2 years. The sample size was calculated using the census method, considering *α* = 0.2, *β* = 0.8, and a mean difference = 0.3.

### 2.2. Inclusion of Participants

The study inclusion criteria based on previous studies, all those who received implant-supported prostheses were included with the following characteristics [4, 10]:

1. Complete demographic information (such as age, gender, and smoking status).
2. Having a systematic disease was not a basis for excluding people unless the implant was one of the contraindications of the treatment.
3. People whose implant treatments were performed by specialized and reliable operators (such as maxillofacial surgeons and periodontists).
4. People who had the information of implant features (manufacturer company) available.
5. People who did not meet the above conditions, such as incomplete information, treatment by nonspecialists, and those who did not consent to participate in the study, were excluded.

### 2.3. Study Variables and Data Collection

Data were collected from participants who met the inclusion criteria, considering various demographic variables such as age, gender, and systemic diseases (including metabolic diseases, cardiovascular diseases, smoking habits, and diabetes). Independent variables such as the implant system used, surgical location, secondary surgeries due to surgical complications (e.g., a fracture of implant fixtures and inferior alveolar nerve (IAN) exposure), early failures (if observed), implant loading time (immediate or delayed), dimensions (length and diameter), type (bone level, tissue level), and the manufacturer of the implant were also taken into account.

### 2.4. DIF

According to previous studies, no specific definition has been provided for DIF [13]. Implant mobility, radiolucency around the implant, and peri-implantitis are considered DIF [14]. However, it is said that DIF is divided into early and late failure [15].

Early failure represents a failure to establish osseointegration of dental implants. Early failure is related to biological factors (not mechanical, etc.) that can be due to peri-implantitis (including soft and hard tissue recession) [15]. Early failure was characterized by the appearance of any of these symptoms within 3–4 months following the surgery [13].

In the present study, according to the mentioned contents, early failure was diagnosed during the study period from the clinical examinations recorded in the patients' files (mobility) and radiographs.

### 2.5. Statistical Analysis

The data collected on the forms was encoded, and SPSS software (version 22, SPSS Inc., Chicago, IL, USA) was utilized to determine the frequency of each variable and perform statistical analysis. Qualitative variables were reported as percentages (%), while quantitative variables were expressed as mean ± standard deviation (SD). The data were analyzed using the Chi-square, Fisher's exact, and Mann–Whitney *U* tests. A *p*-value of 0.05 or lower was deemed statistically significant.

## 3. Results

### 3.1. Patient Characteristics

This study encompassed 983 implants placed in the implant ward of Mashhad Dental School during the study period. The sample consisted of 555 (56.5%) implants in females and 428 (43.5%) in males, with an average age of 49.34 ± 13.67 years (range: 18–93 years).

Additionally, 217 (22.1%) implants were placed in patients with a history of systemic diseases and 22 (2.2%) in participants who smoked. Patients were followed for an average of 13.95 ± 5.88 months (1–38 months).

Early failures occurred in 42 (4.3%) cases. The frequency of early failure was higher in men than women, but the difference was not statistically significant (*p*=0.238). There was also no significant correlation between implant failure and other demographic variables such as age, systemic conditions, and smoking habits (*p*=0.263, *p*=0.388, and *p*=0.316, respectively). Furthermore, the follow-up duration in participants without early DIF was significantly longer (*p* < 0.001) (Table 1).

### 3.2. Implant-Related Parameters

Concerning implant-associated factors, the failure rate was influenced by the implant manufacturer. Specifically, the highest failure rate occurred in Biodenta companies, followed by Medentis and ITI, but the association with early failure was not statistically significant (*p*=0.066). The sandblasted, large grit, acid-etched level exhibited the highest relative frequency of failures; however, there was no significant difference between various implant surfaces and early failures (*p*=0.886). Although there were 10 (6%) implant failures in tissue and 32 (3.9%) in bone, no meaningful association was found between implant-level impression and its failures.  Table 2 presents the implant failure rate according to implant-related factors (Table 2).

### 3.3. Surgery-Related Parameters

The frequency of implant failure rate was examined for the surgery-related variables mentioned (Table 3). The results revealed a significant association between failure rate and incidences during the surgery (*p*=0.005) as well as implant loading time (*p* < 0.001). In other words, early DIF was more likely to occur in patients with records of IAN exposure and implant fixture fractures during the procedure. There was no significant association between the early failure rate and factors such as the planting area, implant dimensions (height and diameter), and type (bone level, tissue level). Slightly more implant failures occurred in the mandible (4.7%) compared to the maxilla (3.8%), but this difference was not significant (*p*=0.511). The study's findings also revealed no association between early DIF and the presence of grafts (*p*=0.351).

## 4. Discussion

Considering the results of the present study, accepting the null hypothesis from all aspects is not possible definitively. Incidence during surgery and implant loading time were among the factors that rejected the study's null hypothesis and showed a significant relationship with DIF (*p*-value <0.05).

Despite the widespread success of dental implants, early fractures continue to occur for various reasons. Early DIF may arise from numerous factors, but the primary causes remain debatable [4, 16]. Consequently, we focused on exploring the relationship between this issue and potential contributing factors. This investigation determined that the rate of early implant failure among 983 implants was 4.3%. In contrast, other studies, such as Derks et al. [17] and Olmedo-Gaya et al. [16], reported rates of 4.4% and 5.79%, respectively. This variation in prevalence could result from differences in sample sizes. The authors propose that early DIF is associated with implant loading time and events occurring during surgery. However, there was no significant association was found between population parameters and implant-related variables.

The reduction of peri-implant pockets can be achieved by using probiotics in conjunction with mechanical debridements, such as scalling and root planing [18, 19]. Butera et al. [20] asserted that the composition of the microbiota plays a role in the progression of peri-implantitis. Nevertheless, applying postbiotic gels and other forms has proven effective in reducing inflammation [18, 19]. This discrepancy may be attributed to inadequate studies on standardized protocols for administering probiotic products and possible functional changes in the inflammatory process.

The impact of age on implant failure remains a contentious issue. In the current study, no significant association with age was observed. Some other authors reached similar conclusions [21, 22], while others noted that older patients are more likely to experience early DIF than younger ones [2, 6, 21, 23–25]. It has been suggested that this is due to declining bone quality and longer healing processes in older patients. In contrast, Manor et al. [26] proposed that DIF occurs more frequently in younger patients. This discrepancy may be attributed to differences in the mean ages of the study populations [4].

Gender was another demographic risk factor examined in this investigation. Consistent with other studies, many researchers found no significant correlation between a patient's gender and the likelihood of DIF [1, 4, 6, 17]. However, several studies identified a statistically significant higher risk in males [16, 21, 25, 27, 28], particularly among those who smoke [7, 21]. In contrast, other studies reported that DIF occurs more frequently in women than men [23]. Although the current research found that DIF was more common in men, the association was not significant. The differences in results may be due to our study's larger sample size.

The influence of systemic disorders and smoking on the success of implant therapy was also assessed. Numerous articles identified a strong connection between smoking habits and early DIF [6, 7, 14, 29, 30]. Additionally, nicotine has been shown in many experiments to reduce implant longevity [31]. Sverzut et al. [32] supported that smoking cannot be considered a risk factor. The present study did not reveal a significant relationship between smoking and early DIF. This discrepancy could be attributed to the small number of smokers (2.2%) in our study population. Buhara and Pehlivan [7] found a significant association between a history of systemic diseases and early DIF; however, the current investigation and other authors reported conflicting findings [2, 22].

During the operation, complications, such as IAN exposure and fractures of the implant fixture, may occur. Implants may also penetrate the maxillary sinus during the procedure. Most studies did not observe a relationship between Schneiderian membrane perforation and implant failure [33]. However, Hernandez-Alfaro et al. [34] held a contrary view. A significant correlation was found between DIF and fractures of the implant fixture. However, insufficient information about this factor was found in similar studies [35].

A higher risk of implant failure was observed in loaded implants [2, 21]. The present study also revealed a significant association between early failure and the implant's loading time.

It is worth noting that fewer studies have investigated the impact of manufacturers on implant failure [26]. The authors did not find a significant difference between these factors.

Atarchi et al. [6] found that implant lengths greater than 11.5 mm had a higher failure rate. However, Manzano et al. [14] concluded that there was a significant relationship between early implant failure and short implant length. In this study, no significant correlation was observed between the length or diameter of implants and DIF.

There are two theories about the implant location and its connection with DIF. Some reports suggest that implants in the mandible are more vulnerable to infectious complications [36] due to their proximity to the alveolar nerve [7, 37]. It has also been suggested that the location of the mandible and the implant fracture have a statistically significant association [31]. In contrast, this study and others reported no differences in implant failure rates between implant sites (anterior, middle, and posterior mandible/maxilla) [21].

It is important to note that DIF is multifactorial, and other parameters, such as surgical traumatic errors, oral hygiene status before and after implant treatment, poor quality and quantity of the patient's bone, and improper choice of implant type, can also be influential [11]. Indeed, the surgeon's experience and skill play a critical role in the success of the implant [6]. Understanding the extent and causes of DIF can assist dentists in making informed decisions regarding treatment settings and alternative treatment options [38].

Analyzing a large sample size is a strength of this study. The primary limitation of this study was its retrospective nature, which relied on available data from archived records, making it impossible to control all variables. Bone quality and quantity were not directly examined in our sample. However, the difference between those with grafts and those without was not significant. We evaluate different regions of the jaw known to have different bone qualities. Therefore, more studies should examine additional risk factors and their importance.

Another limitation that can be mentioned is the lack of examination of some effective factors of implant failure, such as proximity to the sinus floor and hormonal imbalance (especially in women). One of the reasons for its justification is the study's nature. As mentioned, due to the large sample size of the present study, the results of the study may be generalized to a larger population. Notably, the present study is the first epidemiological study of implant failure in Iran.

## 5. Conclusion

The results of this retrospective study suggest a significant relationship between surgical incidents and implant loading time with early DIF. These findings highlight the importance of reducing the incidence of surgical complications to prevent dental implants from failing prematurely. Therefore, the authors suggest that future analyses incorporate these factors. Well-designed future studies are likely to identify additional risk factors.

## Figures and Tables

**Table 1:** Patient characteristics.

Variables	Result	*p*-Value
Gender, *n* (%)		
Female	555 (56.5%)	*p*=0.238
Male	428 (43.5%)
Age, mean ± SD	49.34 ± 13.67 years (range: 18–93 years)	*p*=0.263
Systematic disease, *n* (%)	217 (22.1%)	*p*=0.388
Patients who smoked, *n* (%)	22 (2.2%)	*p*=0.316
Follow-up duration in participants without early DIF, *n* (%)	941 (95.72%)	*p* < 0.001 ^*∗*^

*Note*.  ^*∗*^*p*-Value <0.05 is considered significant.

**Table 2:** Frequency distribution of each of the variables related to implant and surgery mean ± *N*.

Implant factors	*N* (%)	Early failure	*p* Value
Implant manufacturer			0.066^*∗*^
Implantium	249 (25.3%)	9 (3.6)	
DIO	304 (30.9%)	9 (3)	
Dentis	263 (26.8%)	13 (4.9)	
ITI	81 (8.2%)	6 (7.4)	
Biohorizon	30 (3.1%)	0 (0)	
Zimmer	6 (0.6%)	0 (0)	
Biodenta	9 (0.9%)	3 (33.3)	
Swiss	1 (0.1%)	0 (0)	
Medentis	26 (2.6%)	2 (7.7)	
Peed Anyone	9 (0.9%)	0 (0)	
THOMMEN SPI	1 (0.1%)	0 (0)	
Biotem	4 (0.4%)	0 (0)	
Implant surface			0.886^*∗*^
SLA	864 (87.9)	39 (4.5)	
SLA	65 (6.6)	12 (3.1)	
Active	50 (5.1)	1 (2.0)	
RBM	4 (0.4)	0 (0.0)	
Level of implant placement			0.221^*∗∗*^
Tissue level	166 (16.9)	10 (6.0)	
Bone level	817 (83.1)	32 (3.9)	

RBM, resorbable blasting media; SLA, sandblasted, large grit, acid-etched.

**Table 3:** Surgery-related failures.

Surgery factors			
Implant diameter (mm)			0.091^*∗*^
Narrow: 3.7>	160 (16.3)	12 (7.5)	
Regular: 3.7–5	822 (83.6)	30 (3.6)	
Wide: 5<	1 (0.1)	0 (0.0)	
Implant height (mm)			0.338^*∗*^
Short: 8>	13 (1.3%)	1 (7.7)	
Regular: 8–12	955 (97.2%)	40 (4.2)	
Long: 12<	15 (1.5%)	1 (6.7)	
Jaw			0.511^*∗∗*^
Maxilla	470 (47.8%)	18 (3.8)	
Mandible	513 (52.2%)	24 (4.7)	
Planting area			0.092^*∗∗*^
Anterior	284 (28.9)	14 (4.9)	
Middle	339 (34.5)	8 (2.4)	
Posterior	360 (36.6)	20 (5.6)	
Surgical stage			0.362^*∗∗*^
Single	140 (14.2)	8 (5.7)	
Two	843 (85.8)	34 (4.0)	
Placement			0.887^*∗∗*^
Immediate	124 (12.6)	5 (4.0)	
Delayed	859 (87.4)	37 (4.3)	
Existence of grafts			0.351^*∗∗*^
Yes	467 (47.5)	17 (3.6)	
No	516 (52.5)	25 (4.8)	
Incidence during surgery			0.05^*∗*^
Fracture of implant fixtures	1 (0.1)	40 (4.1)	
IAN exposure	2 (0.2)	1 (100)	
None	980 (99.7)	1 (50)	
Loading			<0.01 ^*∗*^
Yes	18 (52.5%)	18 (52.5)	
No	24 (2.5)	24 (2.5)	

*Note*.  ^*∗*^Results obtained from Fisher's exact test.  ^*∗*^ ^*∗*^Results obtained from the Chi-square test.

## Data Availability

Please visit http://implantfailuredatabase.blogfa.com/. For further information, you can communicate with the corresponding author. The authors confirm that all data collected during the study are presented in the manuscript, and no data from the study has been or will be published separately elsewhere.
